# Gene Selection via a New Hybrid Ant Colony Optimization Algorithm for Cancer Classification in High-Dimensional Data

**DOI:** 10.1155/2019/7828590

**Published:** 2019-10-13

**Authors:** Ahmed Bir-Jmel, Sidi Mohamed Douiri, Souad Elbernoussi

**Affiliations:** Laboratory of Mathematics, Computer Science & Applications-Security of Information, Department of Mathematics, Faculty of Sciences, Mohammed V University, Rabat, Morocco

## Abstract

The recent advance in the microarray data analysis makes it easy to simultaneously measure the expression levels of several thousand genes. These levels can be used to distinguish cancerous tissues from normal ones. In this work, we are interested in gene expression data dimension reduction for cancer classification, which is a common task in most microarray data analysis studies. This reduction has an essential role in enhancing the accuracy of the classification task and helping biologists accurately predict cancer in the body; this is carried out by selecting a small subset of relevant genes and eliminating the redundant or noisy genes. In this context, we propose a hybrid approach (MWIS-ACO-LS) for the gene selection problem, based on the combination of a new graph-based approach for gene selection (MWIS), in which we seek to minimize the redundancy between genes by considering the correlation between the latter and maximize gene-ranking (Fisher) scores, and a modified ACO coupled with a local search (LS) algorithm using the classifier 1NN for measuring the quality of the candidate subsets. In order to evaluate the proposed method, we tested MWIS-ACO-LS on ten well-replicated microarray datasets of high dimensions varying from 2308 to 12600 genes. The experimental results based on ten high-dimensional microarray classification problems demonstrated the effectiveness of our proposed method.

## 1. Introduction

In recent years, DNA microarray technology has grown tremendously, thanks to its unquestionable scientific merit. This technology developed in the early 1990s allowed researchers to simultaneously measure the expression levels of several thousand genes [1, 2], These levels of expression are very important for the detection or classification of the specific tumor type. The microarray data is transformed into gene expression matrices, where a row represents an experimental condition and column represents a gene; each value of *x*_*ij*_ is the measure of the level of expression of the *j*^th^ gene in the *i*^th^ sample (see Table 1).

For the cancer classification problem, each line contains information about the class of a sample (the type of cancer). Thus, DNA microarray analysis can be formulated as a supervised classification task [3].

In the cancer classification task, a small number of samples are available, while each sample is described by a very large number of genes. These characteristics of the microarray data make it very likely the presence of redundant or irrelevant genes, which limit the performance of classifiers. Thus, extracting a small subset of genes containing valuable information about a given cancer is one of the principal challenges in the microarray data analysis [4].

Gene selection has become more and more indispensable over the last few years. The main motivation of this selection is to identify and select the useful genes contained in a microarray dataset for distinguishing the sample classes. It also provides a better understanding and interpretation of the phenomena studied. Also, it surpasses the curse of dimensionality in order to improve the quality of classifiers. In general, gene selection methods are divided into two subclasses: wrapper approaches and filter approaches [5]. In wrapper methods, the selection can be seen as an exploration of all the possible subsets, and the principle is to generate a subset of genes and evaluate it afterward. Indeed, the quality of a given subset is measured by a specific classifier. In the aforementioned method (wrapper), the classification algorithm is used several times at each evaluation. Generally, the accuracy according to the final subset of genes is high because of the bias of the process of generating the classifier used. Another advantage is their conceptual simplicity: just generate and test. However, they do not have any theoretical justification for the selection and do not allow us to understand the dependency relationships that may exist between genes. On the other hand, the selection procedure is specific to a particular classifier, and the found subsets are not necessarily valid if we change the classifier. Besides, they typically suffer from a possible overfitting and high computational cost [5, 6]. Also, these approaches become unfeasible because the evaluation of large gene subsets is computationally very expensive [7]. While in filter methods, the final subset is selected based on some gene score functions and significance measures. Unlike wrappers, the selection is independent of the classifier used. The operating principle of these methods is based on the evaluation of each gene individually to assign it a score. The gene selection is performed by selecting the best-ranked genes. Filters are generally less expensive in computing time, so they can be used in the case where the number of genes is very high because of their reasonable complexity. But, the main negative point of these methods is that they do not take into consideration the possible interactions between genes. In the literature, there are several individual gene-ranking methods (filter) such as *t*-test [8], Fisher score [9], signal-to-noise ratio [10], information gain [7], and ReliefF [11].

In wrapper methods, metaheuristics are commonly used to generate high-quality subsets of genes. Examples of classification algorithms used for measuring the quality of each candidate solution include support vector machines (SVMs) and K nearest neighbor (KNN) [12].

The first works on the DNA microarray classification were published at the end of the 1990s [13, 14]. In this context, several researchers have utilized metaheuristic methods and the ACO algorithm for solving the feature selection problem (particularly gene selection), in order to facilitate recognition of cancer cells: ACO [15–20] algorithm, PSO [4, 6, 21–25] genetic algorithm [4, 26, 27], incorporating imperialist competition algorithm (ICA) [28], and binary differential evolution (BDE) algorithm [29].

The ant colony optimization algorithm (abbreviated as ACO) is a population-based metaheuristic [30, 31]. Thanks to its efficiency, it has been used to solve several optimization problems in different fields. In the ACO algorithm, each ant presents a candidate solution to the problem, and the ants build approximate solutions iteratively (step-by-step). The process of constructing solutions can be regarded as a path (between home and food source of ants) on a graph. The choice of the best path by ants is influenced by the quantities of pheromone left in these pathways and a piece of heuristic information that indicates the goodness of the decision taken by an ant.

Thus, metaheuristics find application in solving the gene selection problem which is known to be NP-hard [32, 33]. In the last decade, several researchers have also adopted graph-based techniques to select near-optimal subset of a feature set [34–36].

In this study, we propose a hybrid approach for solving the gene selection problem. Our two-stage proposed approach starts with a first stage in which a new graph-based approach is proposed (MWIS) without using any learning model. In the second stage, a wrapper method based on a modified ACO and a new local search algorithm guided by the 1NN classifier is developed. In this step, the role of 1NN is to evaluate each candidate gene subset generated. The proposed approach has not been previously investigated by previous researchers.

This paper is organized as follows: in Section 2, we present the proposed gene selection method.  Section 3 provides a detailed exposition of the experiments that we have put on ten microarray datasets to evaluate our approach. Finally, we conclude our paper.

## 2. Methods

### 2.1. Graph Theory Approach for Gene Selection

#### 2.1.1. Notations

In this work, we use *X* to denote a dataset (Table 1) of *M* samples =(*x*_1_, *x*_2_,…, *x*_*M*_)*x*_*i*_ ∈ ^*N*^. We use {*g*_1_, *g*_2_,…, *g*_*N*_}, *g*_*i*_ ∈ ^*M*^ to denote the *N* genes vectors. *Y*=(*y*_1_, *y*_2_,…, *y*_*M*_) are the class labels.

Graph theory gives an abstract model to represent the relationships between two or more elements (vertices) into a given system. Let *G*=(*V*, *E*) be an undirected graph where *V* is a nonempty finite set called the set of vertices and *E* is the set of edges. We define a vertex-weighted graph (*G*, *W*) as a graph *G* together with a function *W* (the vertex weighting function) such that *W*(*u*) ∈ *ℝ*^+*∗*^ for all *u* ∈ *V* [37, 38]. The maximum weight independent set (MWIS) is one of the most important optimization problems, thanks to their several domains of application [39], particularly, in the gene selection problem, where we can transform the DNA microarray data into a vertex-weighted graph (gene-similarity graph). In this graph, each gene can be considered as a vertex and their Fisher score as weight of this vertex. The set of edges represents the existence of significant correlation (relationship) between these genes; this relation is nothing but the degree of linear association (Pearson correlation) between the latter. After transforming the DNA microarray data, we try to find the maximum weight independent set. This set of genes will be used in the second stage of our proposed method.

### 2.2. Construction of Gene-Similarity Graph

The construction of gene-similarity graph requires the definition of some statistical notions: starting with the Fisher score to calculate the weight of each vertex (gene).

#### 2.2.1. Fisher Score *F*_*i*_ [9]

It is mainly applied in gene selection as a filter [40]. The Fisher score value of each gene represents its relevance to the dataset; a higher Fisher score means that the gene contributes more information. This information helps to measure the degree of separability of the classes through a given gene *g*_*i*_. It is defined by

(1) $$\begin{array}{c} F_{i}=\frac{\sum_{k=1}^{c}n_{k}{\left(\mu_{k}^{i}-\mu^{i}\right)}^{2}}{\sum_{k=1}^{c}n_{k}{\left(\sigma_{k}^{i}\right)}^{2}}, \end{array}$$

where *c*, *n*_*k*_, *μ*_*i*_^*k*^, and *σ*_*k*_^*i*^ represent, respectively, the number of classes, the size of the *k*‐th class, and mean and standard deviation of *k*^th^ class corresponding to the *i*^th^ gene. *μ*_*i*_ is the global mean of the *i*^th^ gene.

#### 2.2.2. Pearson Correlation Coefficient

The Pearson correlation coefficient is a measure of the strength of the linear relationship between two variables (genes). Let *g*_1_ and *g*_2_ be two random variables, and the correlation coefficient between *g*_1_ and *g*_2_ is defined by

(2) $$\begin{array}{c} \rho_{g_{1},g_{2}}=\frac{\mathrm{cov}\left(g_{1},g_{2}\right)}{\sigma_{g_{1}}\sigma_{g_{2}}}, \end{array}$$

where cov(*g*_1_, *g*_2_) is the covariance between *g*_1_ and *g*_2_, *σ*_*g*_1__ is the standard deviation of *g*_1_, and *σ*_*g*_2__ the standard deviation of *g*_2_.

The correlation coefficient may take on a range of values from −1 to +1. Let (*r*_*ij*_=|*ρ*_*g*_*i*_,*g*_*j*__|) be the absolute value of the correlation between *g*_*i*_ and *g*_*j*_.

Now, we can define the adjacency matrix *A*_*G*_=(*a*_*ij*_)_*N*×*N*_, with zeros on its diagonal to represent (*G*, *W*). Where *a*_*ij*_=1 if (*i*, *j*) ∈ *E* is an edge of *G* and *a*_*ij*_=0 if (*i*, *j*) ∉ *E*. More precisely, a value of 1 represents the existence of a relationship between *g*_*i*_ (row *i*) and *g*_*j*_ (column *j*), while a value of 0 means the nonexistence of this relationship. The creation of *A*_*G*_ requires the definition of the absolute correlation matrix *R*=(*r*_*ij*_)_*N*×*N*_. Based on this matrix, we fill *A*_*G*_; let *r*_0_ be a fixed value in [0,1]; we assume that if *r*_*ij*_ > *r*_0_ then the mutual information between *g*_*i*_ and *g*_*j*_ is high (i.e., the two vertices are adjacent). More exactly, the matrix *A*_*G*_ is filled based on the rule below: for *i* ≠ *j*,

(3) $$\begin{array}{c} a_{ij}=\left\{\begin{array}{cc} 1, & \text{ if }r_{ij}>r_{0},\\ 0, & \text{ otherwise, } \end{array}\right. \end{array}$$

where *r*_0_ ∈ [0,1] is the minimum correlation value for which we consider two genes in relation. The experimental study carried out in our method proves that *r*_0_=0.35 behaves well with the high-dimensional data. For example, if we have a dataset composed of 7 genes {*g*_1_, *g*_2_,…, *g*_7_}, Table 2 shows the corresponding absolute correlation matrix to these data.

For *r*_0_=0.35, the adjacency matrix is given in Table 3.

We define the weight of a vertex *i*(*g*_*i*_), by using the Fisher score: *W*(*i*)=*F*_*i*_. A gene *i* with a high score in the DNA microarray dataset corresponds to a vertex with a high weight in (*G*, *W*). This weight gives important information about the gene relevancy to the data. Indeed, if there are two genes connected by an edge in *G* we prefer the gene which has the best weight. On the basis of the steps defined before, we were able to transform a determined DNA data microarray into a vertex-weighted graph (Algorithm 1).

**Figure 1:**
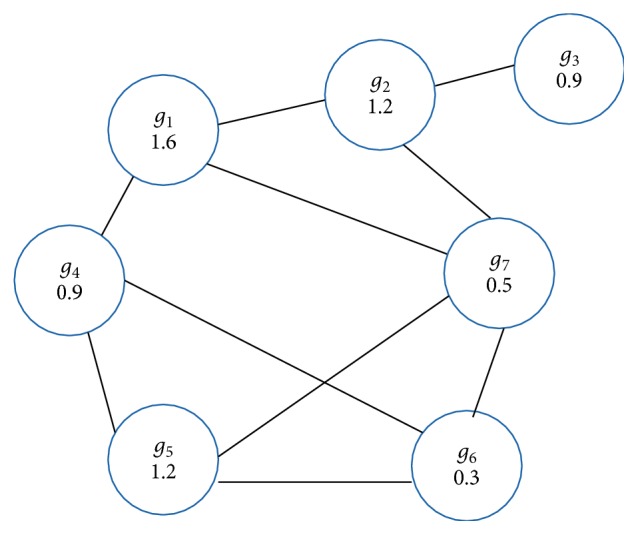
An example of gene-similarity graph.

Figure 1 shows the gene-similarity graph equivalent to the adjacency matrix (Table 3); we associate to each gene (vertex) a weight by using the Fisher score:

In the context of gene selection for cancer classification, the microarray datasets are characterized by a very large number of genes. The application of an evolutionary algorithm such as ACO directly without passing by a preprocessing step is highly expansive. This is where filter methods become so useful in order to extract a subset of possibly informative genes, and then the evolutionary metaheuristic is applied to select the near-optimal subset of genes [19]. As examples, generalized Fisher score, ReliefF, and BPSO are combined in [6], an information gain filter and a memetic algorithm in [41], chi-square statistics and a GA are used in [26], information gain and improved simplified swarm optimization in [42].and ReliefF, mRMR (minimum redundancy maximum relevance), and GA in [11]. Zhao et al proposed a hybrid approach by combining the Fisher score with a GA and PSO [40]. In order to overcome the disadvantages of filter methods, we propose an efficient approach based on graph theory techniques to select the first subset. This method takes into account possible interactions between genes.

### 2.3. Gene Selection Based on the Maximum Weight Independent Set

Let (*G*, *W*) be a vertex-weighted undirected graph, where *V* is the set of its vertices, *E* is the set of edges, and *W* is the vertex weighting function. For each *v* ∈ *V* we define *N*_*G*_(*v*) the neighborhood of *v*, i.e., *N*_*G*_(*v*)={*u* ∈ *V* : *u* ≠ *v*, (*u*, *v*) ∈ *E*}. A subset *I* ⊆ *V* is an independent set of *G* if there are no two adjacent vertices in *I* (i.e., connected by an edge). The MWIS is the independent set with the maximum weight (the weight of a subset of vertices in *V* is defined as the sum of the weights of the vertices in this subset [43]).

We remark that in filter methods for gene selection based on the rank of genes, the correlation between the selected genes is not considered. This implies the selection of subsets with a high level of redundancy that penalizes the classification performances; on the other hand, these methods eliminate the genes with a low individual score, ignoring the possibility that they can become highly relevant when combined with other genes [44]. This motivates us to propose a graph-based approach to overcome these problems. In the first stage of our method, we consider the gene selection problem as the search for the maximum weight independent set in the gene-similarity graph (*G*, *W*). The choice of this subset is justified by two arguments: First, the term maximum weight can be translated in the context of gene selection as selecting a subset of genes with maximum relevance. Second, the notion of independent ensures the choice of a subset with minimum redundancy; i.e., in this subset, there are no two genes with high correlation. In addition, this subset can contain genes with a low score. Therefore, the proposed method in this stage gives a good subset of genes for applying an evolutionary algorithm such as ACO.

The MWIS into a given graph is an NP-hard problem [45], and since in our case the gene-similarity graph is large (several thousands of vertices and edges), then it is impossible to find an exact solution to our problem in a reasonable time. For this, we propose a greedy algorithm (heuristic) to quickly obtain an approximate solution. The main lines of this algorithm are presented in Algorithm 2.

**Figure 2:**
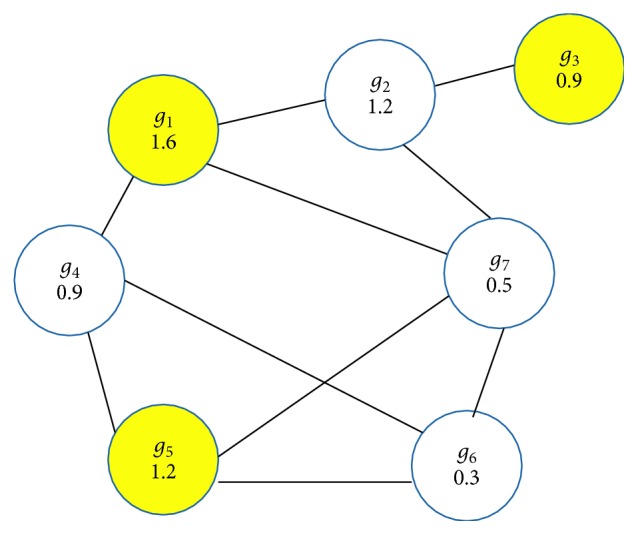
Example of maximum weight independent set.

We illustrate the execution of our greedy algorithm (Algorithm 2) on the graph from Figure 1 formed by {*g*_1_, *g*_2_,…, *g*_7_}. In the first iteration, we select the best gene *g*_1_ (*W*(*g*_1_)=1.6), then we remove their neighborhood {*g*_2_, *g*_4_, *g*_7_}, and in the next iteration we choose the best gene *g*_5_ in the second graph composed by {*g*_3_, *g*_5_, *g*_6_}. In the last iteration, we have only one gene to choose *g*_3_. Then *I*={*g*_1_, *g*_3_, *g*_5_} (Figure 2) is an approximate maximum weight independent set, and we can notice that our greedy algorithm gives the exact MWIS for this example.

### 2.4. Ant Colony Optimization for Gene Selection

ACO is one of the algorithms based on swarm intelligence. It was introduced as a method for solving optimization problems in the early 90s by Dorigo et al. [30, 31] and developed after in [46, 47]. Initially, ACO was designed to solve the traveling salesman problem by proposing the first ACO algorithm: “Ant System” (AS) [48]. Subsequently, other applications that were considered early in the history of ACO such as quadratic assignment [49], sum coloring [50], vehicle routing [51], constraint satisfaction [52], and gene selection [15–17, 19, 20].

The ACO algorithm is inspired by the social behavior of ants. The artificial ants used in the ACO can cooperate with each other (by exchanging information via pheromones) to solve difficult problems; this is performed by building approximate solutions iteratively (step-by-step). The feasible solutions can be regarded as a path between home and food source of ants. The method of choice of this last path is detailed in the next subsections.

#### 2.4.1. ACO for Gene Selection

**Figure 3:**
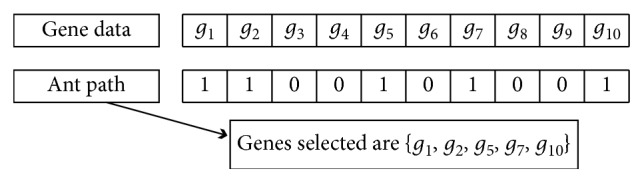
An illustrated example with generated subset and path representation.

Denote the *p* genes as {*g*_1_, *g*_2_,…, *g*_*p*_} to adopt the ACO for gene selection problem, and a novel ACO is proposed; the path of each ant from the nest to food is coded as a *p*‐dimensional binary string where each bit of the pathway is attached to a gene; the selection of the pathway “1” means that gene has been chosen. On the other hand, a pathway “0” indicates that the gene is not selected in the final subset. Suppose that *p* is 10, the coding of our modified ACO is presented and explained in Figure 3.

**Figure 4:**
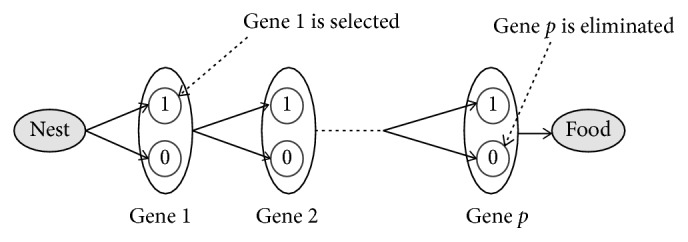
The gene selection procedure of modified ACO.

The ants seek to find the best path that maximizes the accuracy and minimizes the number of selected genes.  Figure 4 describes the gene section procedure proposed on our ACO. Each ant starts from the nest to the food source with the aim to find the best path (best subset of genes). The building of this path is done step-by-step; in each step *i*, the ant decides to add the gene *i* to the candidate subset of genes or not, based on the pheromone and heuristic information assigned to this gene (Figure 4). The ant terminates its tour in *p* steps and outputs a subset of selected genes as it reaches the food source.

As indicated previously, the task of each ant is to construct a candidate subset of genes using heuristic information and pheromone; this is performed via a probabilistic decision rule. We compute the probability of selecting a pathway as below:

(4) $$\begin{array}{c} p_{ij}=\frac{\left(\tau_{ij}^{\alpha }\right)\left(\eta_{ij}^{\beta }\right)}{\left(\tau_{i0}^{\alpha }\right)\left(\eta_{i0}^{\beta }\right)+\left(\tau_{i1}^{\alpha }\right)\left(\eta_{i1}^{\beta }\right)},\;\left(i=1,2,\ldots ,p;\;j=1,0\right), \end{array}$$

where *i* represents the *i*^th^ gene, *j* takes the value 1 or 0 to denote whether the corresponding gene has been selected or not, *τ*_*ij*_ is the pheromone intensity that indicates the importance of the selection of the *i*^th^ gene, and *η*_*ij*_ represents the heuristic reflecting the desirability of the selection of this gene or not. *α* and *β* are two parameters controlling the relative importance of the pheromone intensity versus visibility; with *α*=0, only the visibility (heuristic information) of the gene is taken into account, and the ants will decide to select or not a given gene based just on *η*_*ij*_. Since the previous research experience is lost, therefore there is no cooperation between ants in this case. On the contrary, with *β*=0, only the trail pheromone trails play. To avoid too rapid convergence of the ACO algorithm, a compromise between these two parameters is necessary to ensure the diversification and intensification of the search space.

#### 2.4.2. The Heuristic

The choice of a good heuristic, which will be used in combination with the pheromone information to build solutions, is an important task in the ACO implementation [53]. In our ACO, this heuristic is used to indicate the quality of a gene based on a scoring algorithm.

For a given ant, the heuristic information *η*_*i*1_ is the desirability of adding the gene *i* to the subset of selected genes. We define this quantity based on the Fisher score *F*_*i*_ (1) which measures the quality of this gene and the number of genes selected by the ant before arriving at gene *iN*_s_. *η*_*i*1_ is calculated as follows:

(5) $$\begin{array}{c} \eta_{i1}=\frac{F_{i}}{1+N_{\text{s}}}. \end{array}$$

For the value of *η*_*i*0_, we combine the mean of the scores of Fisher of all genes and *N*_s_. This means that the ants tend to choose the small subsets of genes that have high relevance:

(6) $$\begin{array}{c} \eta_{i0}=\frac{1}{p\left(1+N_{\text{s}}\right)}\overset{k=p}{\underset{k=1}{\sum }}F_{i}. \end{array}$$

#### 2.4.3. Updating the Pheromone Trail

The goal of the pheromone update is to increase the pheromone values associated with good solutions while reducing those associated with bad ones.

The updates of pheromones are made in two stages, a local update and a global update.

Once the ant *k* has finished the built of its path, the pheromone in all of the pathways will be updated. The updated formula is described below:

(7) $$\begin{array}{c} \tau_{ij}\;⟵\;\left(1-\rho_{\text{loc}}\right)\tau_{ij}+\rho_{\text{loc}}\Delta \tau_{ij}, \end{array}$$

where *ρ*_loc_ is the local pheromone evaporation coefficient parameter (0 < *ρ*_loc_ < 1) which represents the evaporation of trail and Δ*τ*_*ij*_ is the amount of pheromone deposited by the ant *k*; in our ACO, it is given by

(8) $$\begin{array}{c} \Delta \tau_{ij}=\left\{\begin{array}{cc} {\text{CA}}_{1\text{NN}}\left(S\right)\;*\;\left(\lambda -\frac{\#\text{ genes }}{p}\right), & \text{if the ant }k\text{ uses the pathway }j\text{ when it arrived at gene }i,\;\\ 0, & \text{otherwise,} \end{array}\right. \end{array}$$

where *S* is the candidate solution created by the ant, CA_1NN_ is the *r*-fold cross-validation classification accuracy of 1NN classifier (nearest neighbor) based on *S*, #genes is the number of selected genes in *S*, and *λ* is a parameter that indicates the importance of the number of selected genes in *S* (1 ≤ *λ*).

At each iteration *T*, after all ants finish their traverses, a global update of pheromone quantities is made for all pathways chosen by the best ant (the best candidate solution) during the iteration *T*.

The global update is carried out as follows:

(9) $$\begin{array}{c} \tau_{ij}\;⟵\;\left(1-\rho_{\text{glob}}\right)\tau_{ij}+\rho_{\text{glob}}\Delta \tau_{ij}\left(T\right), \end{array}$$

where *ρ*_glob_ is the global pheromone evaporation coefficient parameter and Δ*τ*_*ij*_(*T*) is the amount of pheromone deposited by the best ant during the iteration *T* given by Chiang et al. [15].

To avoid stagnation of the search, the range of possible pheromone trails is limited to an interval [*τ*_min_; *τ*_max_].

### 2.5. Fitness Function

In order to guide our novel ACO towards a high-quality subset of genes, we need to define a “fitness function” *f*. The quality of a candidate subset can be measured by combining the number of genes into this subset (size) and the classification accuracy using a specific classifier, and in gene selection the aim is to maximize the accuracy and minimize the number of genes used.

The estimation of the classification accuracy is measured by a given classifier using the cross-validation rule. In this study, we use the K-nearest neighbor classifier (KNN).

#### 2.5.1. K-Nearest Neighbor (KNN)

The KNN method is a supervised learning algorithm and was introduced by Fix and Hodges in 1951 [54]. It is based on the notion of proximity (neighbor) between samples for making a decision (classification) [55].

In order to determine the class of a new example, we calculate the distance between the new one and all testing data, and finally the classification is given by a majority vote of its *K* neighbors. The neighbors are determined by the Euclidean distance which is defined as follows:

(10) $$\begin{array}{c} D\left(X_{1},X_{2}\right)=\sqrt{\overset{p}{\underset{i=1}{\sum }}{\left(x_{1i}-x_{2i}\right)}^{2}}. \end{array}$$

In our proposed method, we use the 1NN classifier, which is a particular case of KNN (with *K*=1). Let *X* be a new sample to classify and *T* a sample from the training data, then the class of *X* is determined as below:

(11) $$\begin{array}{c} \text{Class}\left(X\right)=\text{Class}\left(\arg\min\left(D\left(X,T\right)\right)\right). \end{array}$$

Note that, the genes into gene expression data had different scales, and the KNN classifier is influenced by the measure of distances between samples. Therefore, we modify our 1NN by normalizing the training data to transform them to a common scale. This transformation is carried out based on the mean and the standard deviation of each gene, and the latter values are used for the scaling of the test data.

#### 2.5.2. Objective Function

The fitness value of a candidate solution *S* in our ACO is calculated as follows:

(12) $$\begin{array}{c} f\left(S\right)=w_{1}\;*\;{\text{CA}}_{1\text{NN}}\left(S\right)+\left(1-w_{1}\right)\;*\;\left(1-\frac{\#\text{genes}}{p}\right), \end{array}$$

where *w*_1_ is a weight coefficient in [0,1] that controls the aggregation of both objectives (maximizing the predictive accuracy and minimizing the number of genes), #genes is the number of selected genes in *S*, and *p* is the total number of genes.

Mention that“CA_KNN_” is nothing but the average cross-validation classification accuracy calculated by the KNN classifier, using leave-one-out-cross-validation (LOOCV) [56], in which we divide our dataset into *M* nonoverlapping subsets (*M* tissue samples). At each iteration, we train our KNN classifier on (*M* − 1) samples based on the selected genes, and we test it on the remaining sample. The“CA_KNN_” associated to LOOCV is calculated based on the rule below:

(13) $$\begin{array}{c} {\text{CA}}_{\text{KNN}}=\frac{\text{the number of correctly predicted samples}}{M}. \end{array}$$

### 2.6. Local Search

The local search algorithm is used to improve the solutions given by ants and provide good solutions within a reasonable time. With this aim, we are inspired by the framework proposed in [57], in which a local search based on the filter ranking method is used to solve the feature selection problem.

Given a candidate solution generated by an ant, we define *X* and *Y* as the subset of selected and eliminated genes, and *X* and *Y* both are ranked using Fisher score, respectively. We further define two basic operators of the local search algorithm:

- **Add**: select gene from *Y* based on its ranking and add it to *S*
- **Del**: select gene from *X* based on its ranking and remove it from *S*

The selection of the gene *i* from *Y* to move it to *S* by **Add** operator in our proposed method is based on the Roulette wheel developed by Holland [58]. Let *Y*={*g*_1_, *g*_2_,…, *g*_*n*_1__} and {*F*_1_, *F*_2_,…, *F*_*n*_1__} be its Fisher score values. Then the selection probability *P*_*i*_ for gene *g*_*i*_ is defined as follows:

(14) $$\begin{array}{c} P_{i}=\frac{F_{i}}{\sum_{j=1}^{j=n_{1}}F_{j}}. \end{array}$$

Similarly, for the operator **Del**, we define the probability of selecting a gene *g*_*i*_ of *X*={*g*_1_, *g*_2_,…, *g*_*n*_2__} to remove it from *S* with a probability defined by:

(15) $$\begin{array}{c} P_{i}=\frac{\bar{F_{i}}}{\sum_{j=1}^{j=n_{2}}\bar{F_{j}}}, \end{array}$$

where $\bar{F_{j}}=\max\left(F_{1},\ldots ,F_{n_{2}}\right)-F_{j}$, for *j*=1,…, *n*_2_, and {*F*_1_; *F*_2_,…, *F*_*n*_1__} are the Fisher score values of {*g*_1_, *g*_2_,…, *g*_*n*_2__}.

Based on the probabilities defined before, we can remark that **Add** operator prefers the genes with the high score to add to *S*, on the other hand, **Del** operator prefers the genes with the low score to remove from *S*.

Our local search algorithm (Algorithm 3) is characterized by the number maximal of **Add***n*_add_ and **Del***n*_del_ operations, and it_max_ the maximal number of consecutive iterations without improvement in the best solution. In addition, this local search algorithm is general and efficient, for example, if we fix *n*_add_ at 0, the local search algorithm becomes a **backward** generation, in which we try to remove the not relevant genes at each iteration.

### 2.7. Proposed Method for Gene Selection (MWIS-ACO-LS)

**Figure 5:**
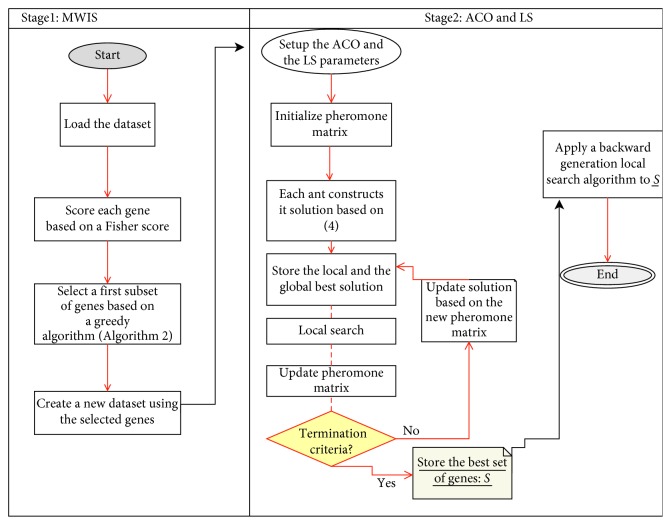
Flowchart of our proposed approach for gene subset selection in DNA microarray data.

Our hybrid method for solving the gene selection problem is based on combining filter and wrapper approaches. This is carried out taking advantage of the low computing time in filters (MWIS) and the high quality of the subsets provided by the wrapper methods (ACO and LS). The overall process of MWIS-ACO-LS can be seen in Figure 5.

The process begins by transforming the initial dataset into a vertex-weighted graph (Algorithm 1), where we search the MWIS, which is well-known as an NP-hard problem, so we have proposed a greedy algorithm (Algorithm 2) to find a near-optimal set of vertices (representing genes in our problem). The subset of genes selected in the later stage is taken as input into the second stage of selection, which used an evolutionary algorithm (ACO), combined with a local search algorithm to select the minimum number of genes that gives the maximum classification accuracy for the 1NN classifier. In this stage, artificial ants cooperate to build a high-quality subset of genes based on the transition rules already presented in Section 2. Also, a local search (Algorithm 3) is proposed to help the ants to achieve good results in a reasonable time. The pseudocode of our proposed method is presented as follows.

### 2.8. Complexity Analysis of MWIS-ACO-LS

Suppose that *N* is the number of the original genes and *M* is the number of samples. Our method is divided into three principal stages:

  Stage 1: In the first step (Algorithm 1), the weight values of the genes are evaluated using the Fisher score, thus the time complexity is *O*(*NM*). Moreover, the absolute correlation values between each pair of genes are computed, so the time complexity is *O*(*N*^2^*M*). And finally, for the filling of the adjacency matrix *A*_*G*_ (implicitly the construction of gene-similarity graph), the time complexity is *O*(*N*^2^). In the second step of this stage (Algorithm 2), the weight of each vertex is already defined, and then we can conservatively assume that in each iteration we remove only the vertex itself to get a time complexity of *O*(*N*). Therefore, the overall time complexity of this stage (MWIS) is *O*(*NM*+*N*^2^*M*+*N*^2^+*N*)=*O*(*N*^2^*M*).
  Stage 2: First, we mention that *p* represents the number of selected genes in the first stage; generally (*p* ≪ *N*).
  In this stage, the fitness of each candidate subset of genes is calculated using LOOCV (leave-one-out-cross-validation) and the 1NN as a classifier equation (13). Let us analyze now the complexity of fitness calculation using 1NN (LOOCV): we compute the distance between the single sample of the testing set and each training set sample, requiring *O*(*p*(*M* − 1)), this process is repeated *M* times, so the fitness calculation need *O*(*p*(*M* − 1)*M*).
  In each iteration of our ACO, each ant from the *m* ants starts from *g*_0_ to *g*_*p*_ passing by all p genes, then the construction of a path by an ant as *O*(*p*), and each path is evaluated by LOOCV. This process is repeated *n*_max_ times by the *m* ants, in addition, updating the pheromone values has *O*(*pn*_max_); therefore, the overall computational complexity of ACO without local search is *O*(*pn*_max_+*pn*_max_*mp*(*M* − 1)*M*)=*O*(*n*_max_*mp*^2^(*M* − 1)*M*).
  Concerning the local search algorithm, for the LS used in the second stage (line 20 Algorithm 4) repeated *n*_max_ times, the complexity time is *O*((*n*_add_+*n*_del_)it_max_*n*_max_*p*(*M* − 1)*M*).
  Generally, (*n*_add_+*n*_del_)it_max_≃*m*, so the total complexity time of the second stage is *O*(*n*_max_*mp*^2^(*M* − 1)*M*).
 
**Stage 3**: **For** the last stage (line 24 Algorithm 4), the complexity of the backward generation is *O*(it_max_*n*_del_*p*(*M* − 1)*M*).

Consequently, the total time complexity of the proposed method MWIS-ACO-LS is *O*(it_max_*n*_del_*p*(*M* − 1)*M*+*n*_max_*mp*^2^(*M* − 1)*M*+*N*^2^*M*)=*O*(*n*_max_*mp*^2^(*M* − 1)*M*+*N*^2^*M*).

## 3. Experimental Studies

This section presents the performance of our proposed approach (MWIS-ACO-LS) on ten well-known gene expression classification datasets, and we compare our results with those of the state-of-the-art. Furthermore, the characteristics of the used datasets, the parameter settings, and the numerical results will be described in the following sections.

The implementation of the proposed approach (MWIS-ACO-LS) is performed using Matlab R2017a.

As far as the KNN classifier is concerned, we have chosen a predefined function in Matlab. Similarly for the SVM classifier [59, 60] used in the comparison a predefined binary linear classifier was chosen. In addition, we have developed a multiclass SVM classifier based on the one-against-all strategy.

Concerning the logistic regression (LR) classifier we have regularized the cost function by two penalties, the first is lasso (*L*_1_) and the second is the elastic net regularization. The minimizing the cost functions used on LR − *L*_1_ and **LR-Elasticnet** is assured by the stochastic gradient descent (SGD) algorithm implemented in the **Scikit-learn** package [61]. Experimental initial parameters are given in Table 4.

Additionally, in this study, we use leave-one-out-cross-validation (LOOCV) to measure the quality of the candidate subsets of genes and for comparing our results with the other works.

### 3.1. Environment

To evaluate our approach, we have chosen ten datasets (DNA microarray) concerning the recognition of cancers [62], which are publicly available and easily accessible. In addition, these datasets are used in several supervised classification works, particularly in the papers using in the “Comparison with state-of-the-art algorithms” section.

All datasets used are described in Table 5. The latter datasets have a multitude of distinguishing characteristics (number of genes, number of samples, and binary classes or multiclasses). The number of samples in some datasets is small (Brain_Tumor2, 9_Tumors, etc.), while others have a higher number (Lung_Cancer, 11_Tumors, etc.). Also, some of them have binary classes (Prostate_Tumor, DLBCL) while others have multiclasses (Leukemia1, Lung_Cancer, etc.). And as our proposed method is designed for the high-dimensional microarrays, all these datasets are characterized by thousands of genes ranging from 2308 to 12600.

### 3.2. Parameters

We note that our approaches have been run on an Acer Aspire 7750g laptop with Intel Core I5 2.30 GHz processor and 8 GB RAM, under system running Windows 7 (64 bit).

Several tests were carried out in order to obtain an appropriate parameterization; indeed, a set of initial values for the parameters were fixed, and then we change the value of one parameter for different runs until the solutions could not be ameliorated. The process of adjustment was repeated for each parameter until the solutions could not be improved. This process is carried out based on one dataset of cancer classification. Table 5 represents the parameters of the proposed approach.

### 3.3. Results and Comparisons

Firstly, in order to limit the search space and accelerate the speed of convergence of our proposed approach, the first subset of genes was selected based on a graph-theory algorithm for gene selection (MWIS), and then a modified ACO-1NN coupled with a local search algorithm was applied to find more excellent subset of genes. The quality of a candidate subset is measured by the performance of the KNN classifier obtained using LOOCV and the size of this subset.

The objectives of the experiments carried out on the ten datasets of (DNA microarray) are as follows: to test the effect of gene selection on the improvement of the classification accuracy and to validate the proposed method and verify its effectiveness.

Given the nondeterministic nature of our approach and the SGDlogistic classifier, ten independent runs were performed for each dataset to obtain a more reliable result.

Table 6 shows the results obtained using a new graph-based approach (MWIS) for gene selection, and then, using the MWIS-ACO method where we apply to the subset selected by the MWIS and ACO algorithm, and finally using our new improved method MWIS-ACO-LS, where the ACO is coupled with the local search (LS) method. The classification accuracy in MWIS, MWIS-ACO, and MWIS-ACO-LS is calculated using the 1NN classifier, on the other hand, these methods are compared with SVM, 1NN, and SGDlogistic penalized classifiers without selection to demonstrate the usefulness of our selection approach. We analyze our results in three ways:

- The classification accuracy
- The number of genes used in the classification
- The execution time

## 4. Discussion

**Figure 6:**
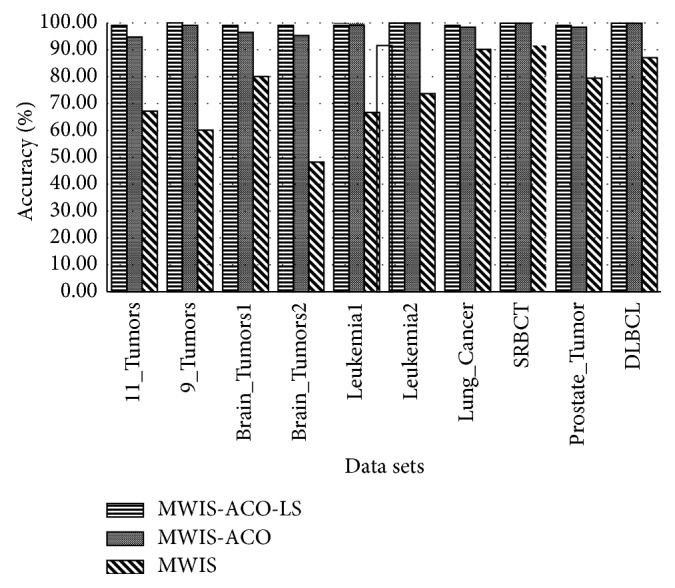
Comparison of the classification accuracy between MWIS, MWIS-ACO, and MWIS-ACO-LS (for MWIS-ACO and MWIS-ACO-LS we take the average solution).

**Figure 7:**
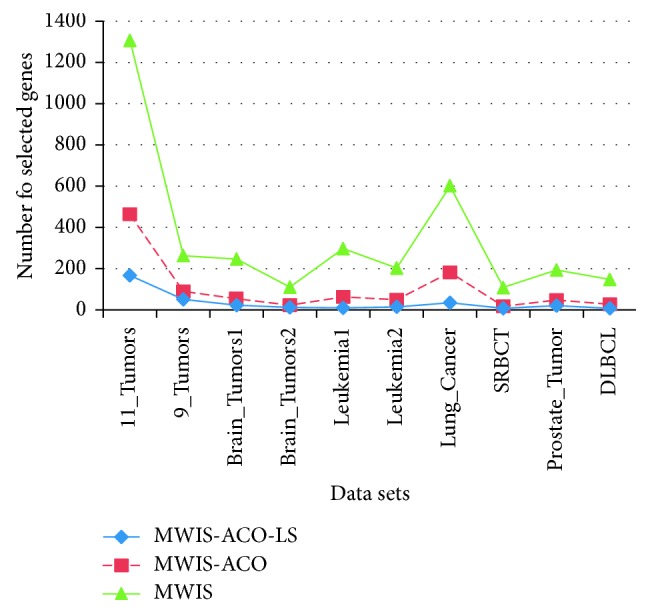
Comparison of the number of genes used in the classification between MWIS, MWIS-ACO, and MWIS-ACO-LS (for MWIS-ACO and MWIS-ACO-LS we take the average solution).

First of all, we start by the execution time analysis of our proposed methods. We can remark that the execution time is appropriate to the complexity analysis; in the filter-based approach MWIS, the execution time is low, but the accuracy is not good since the selection is independent to the classifier. While in MWIS-ACO and MWIS-ACO-LS the execution time is important because of the nature of the wrappers method used and the use of the 1NN classifier at each evaluation, but the classification accuracy is high. Now passing to the analysis of the different stages of our proposed method MWIS-ACO-LS (from Figures 6 and 7 and Table 6), we can remark that the role of the ACO is to improve the classification accuracy and reduce the number of genes used. In addition, the local search has a primordial role in the refinement of the candidate solutions provided by the ants by reducing the number of genes, while retaining the classification accuracy proved by ants.

**Figure 8:**
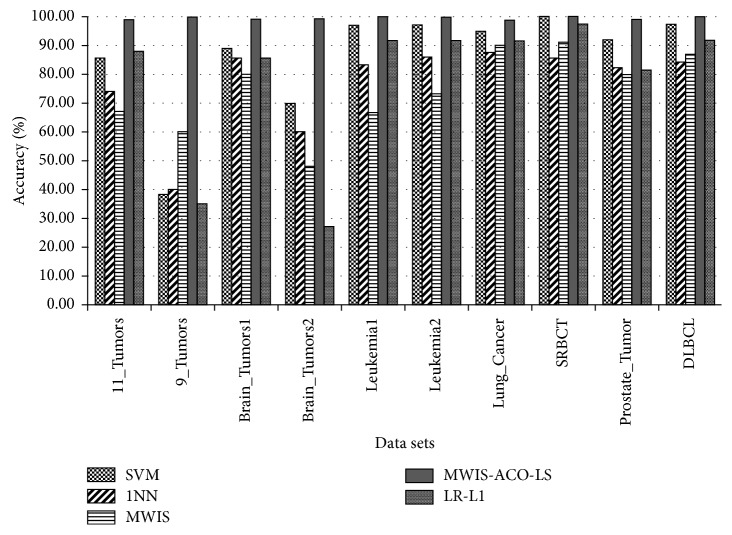
Comparison of the average classification accuracy between the five methods (for MWIS, MWIS-ACO-LS, and LR − *L*_1_ we take the average solution).

The proposed approach (MWIS-ACO-LS) derives its effectiveness from the remarkable improvement in the classification accuracy and the reduction of the number of the genes used in the classification (shown in bold in Table 6), in all datasets (Figure 8).

The “MWIS,” “MWIS-ACO,” and “MWIS-ACO-LS” methods select a reduced subset of informative genes compared to the original subset of genes in the datasets.

From Table 6 and Figure 8, it can be observed that MWIS overcomes the results obtained by the 1NN classifier for “9_Tumors,” “Lung_Cancer,” “SRBCT,” and “DLBCL” datasets which is amazing because the role of the MWIS algorithm is just to find a candidate subset of genes to apply our modified ACO. That subset can contain weak genes and the process of the selection in this method is independent of the classifier used.

Based on the experiments and the application of our approach on ten dataset concern the cancer recognition, we can observe that the proposed method (MWIS-ACO-LS) outperforms all four algorithms in terms of classification accuracy and the number of genes used in the classification. The improvement in performance is more significant for the 9_Tumors; we are passed from a classification accuracy less than 60% to a perfect classification using just 40. So, we can conclude (from Table 6 and Figure 8) that MWIS-ACO-LS can successfully select a small subset of genes which can obtain a high classification accuracy. For all datasets, the “MWIS-ACO-LS” approach has reached a great classification accuracy, more exactly, a classification greater than 99.42% using only a small subset of genes from the original genes. In addition, MWIS-ACO-LS gave a perfect accuracy of 100% for the majority of datasets: (9_Tumors, Brain_Tumor1, Brain_Tumor2, Leukemia1, Leukemia2, SRBCT, Prostate_Tumor, and DLBCL) using just 5 genes for Leukemia1, and 6 genes for SRBCT and DLBLC dataset.

However, with regard to the MWIS approach based on some graph theory principles, we remark that the subset of genes selected by this method gives acceptable classification accuracy according to the number of genes used. This goes back to the procedure used for the construction of this subset in which we give to the genes with the low score the opportunity to be present. As detailed in Section 2, our two-stage proposed method MWIS-ACO-LS starts by selecting a small initial subset of genes that contains the major information in the first stage using MWIS, and then we call our modified ACO combined with a local search algorithm. In this second stage, our algorithm tries to find the smallest subset of genes that give the highest classification accuracy, and Table 6 shows how the second stage plays a crucial role in the increase of the classification for all dataset, especially (Brain_Tumor2, 9_Tumors, 11_Tumors, and Leukemia1) where the results are significantly different (great improvement in the classification accuracy).

**Figure 9:**
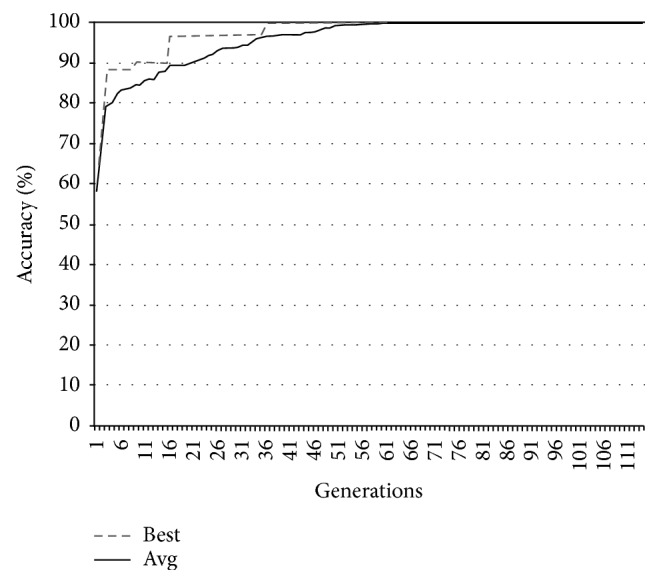
Comparison of the evolution of the classification accuracy for “9_Tumors.”

**Figure 10:**
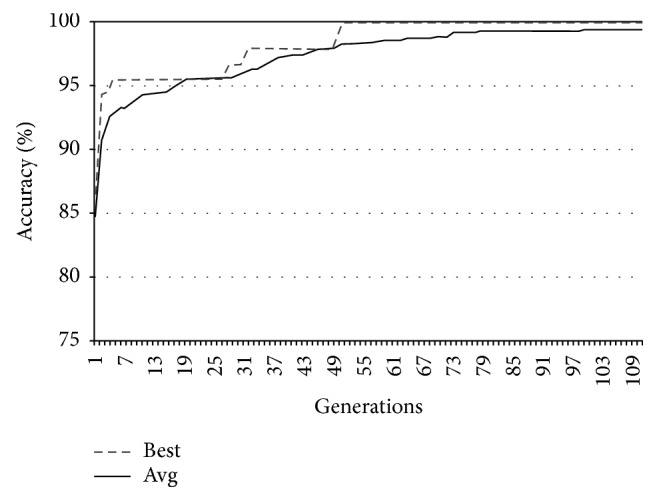
Comparison of the evolution of the classification accuracy for “Brain_Tumor1.”

**Figure 11:**
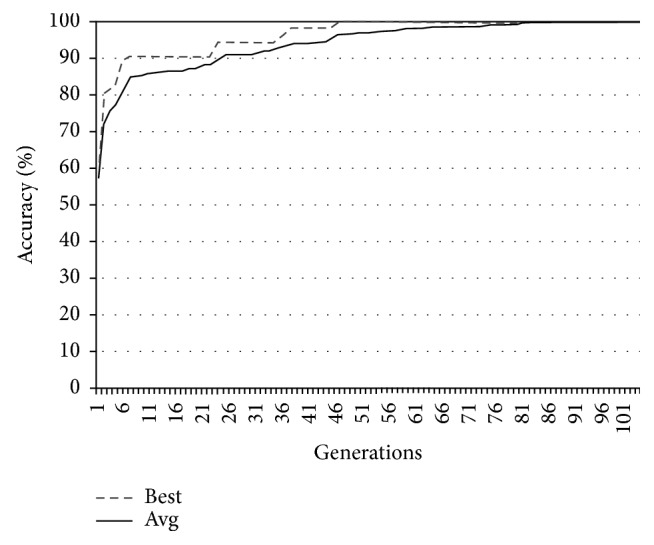
Comparison of the evolution of the classification accuracy for “Brain_Tumor2.”

**Figure 12:**
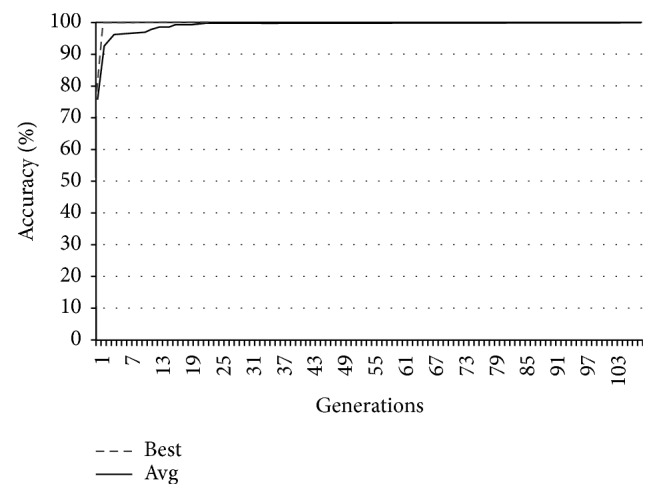
Comparison of the evolution of the classification accuracy for “Leukimia1.”

In Figures 9–12, the abscissa axis expresses the number of generations in the second stage of MWIS-ACO-LS, and the ordinate axis expresses the classification accuracy of the best candidate solution during each iteration. This is done for the average of all solutions and the best solution found for the datasets “9_Tumors, Brain_Tumor1, Brain_Tumor2, and Leukemia1.” These figures clearly show that the use of our modified ACO and the local search algorithm play a crucial role in the amelioration of the classification accuracy. As we can remark in these figures the difference between the best solution and the average solution is not great. Therefore, MWIS-ACO-LS possesses a faster convergence speed and achieves the optimal solution rapidly.

**Figure 13:**
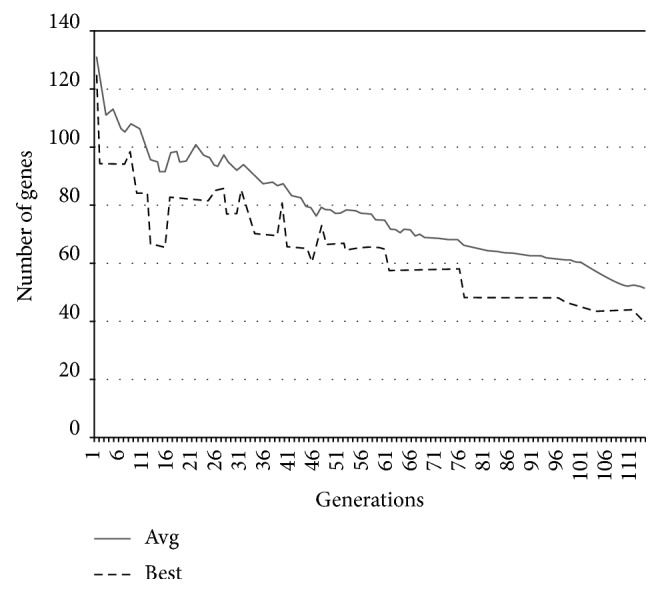
Comparison of the evolution of the number of genes used for “9_Tumors.”

**Figure 14:**
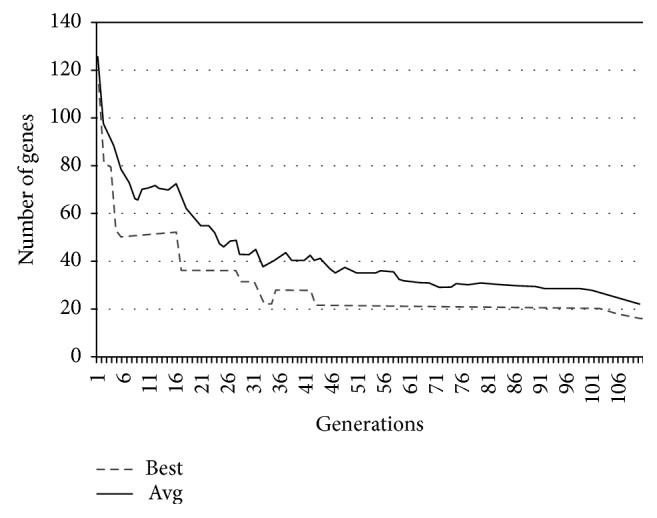
Comparison of the evolution of the number of genes used for “Brain_Tumor1.”

**Figure 15:**
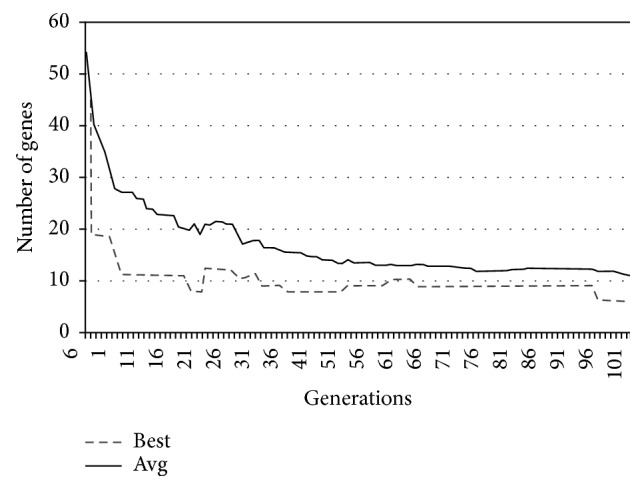
Comparison of the evolution of the number of genes used for “Brain_Tumor2.”

**Figure 16:**
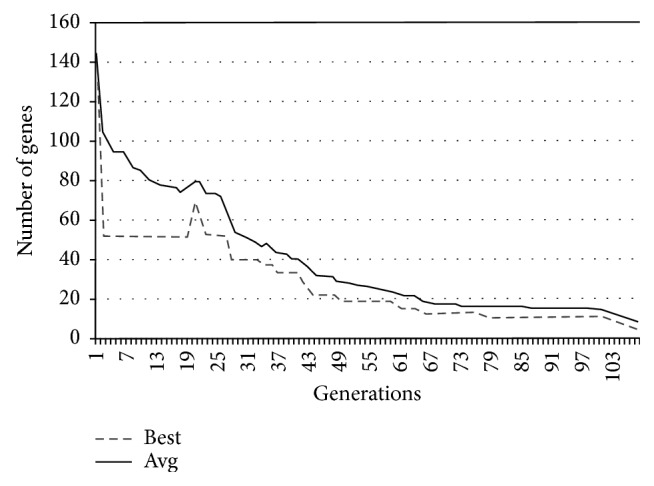
Comparison of the evolution of the number of genes used for “Leukimia1.”

In Figures 13–16, we show the evolution of the number of genes selected on the ordinate axis relative to the number of generations (the abscissa axis) for the “9_Tumors, Brain_Tumor1, Brain_Tumor2, and Leukemia1” datasets. These figures illustrate the role of our wrapper algorithm based on ACO in reducing the number of genes. Moreover, the second stage of our proposed approach based on the modified ACO and the local search algorithm plays a key role in increasing the classification accuracy and minimizing the number of genes used. Indeed, the ACO aims to identify the near-optimal subset of candidate genes, called the best ant, during each iteration that maximizes the objective function, and once the subset in question is found, our local search algorithm is called to ameliorate the accuracy or reduce the number of genes used while retaining the classification accuracy found previously. After 100 generations of the ACO algorithm, we apply a backward local search algorithm to reduce the number of genes used in the last found best solution (Figures 13–16). Thereafter, statistical analysis has been performed using the Kruskal–Wallis statistical test to evaluate our results and test the significance of the difference in the results (accuracy) obtained by our approach.

**Figure 17:**
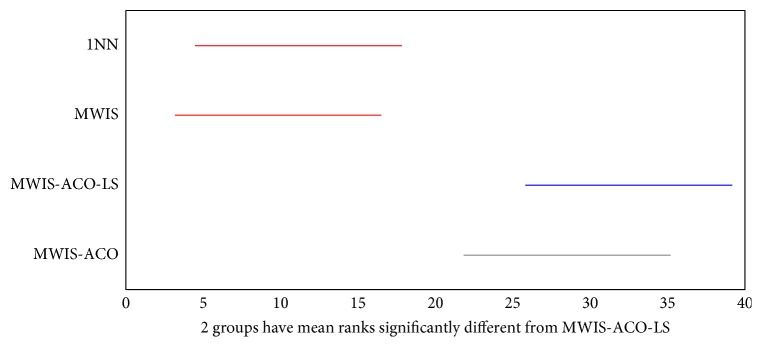
The result of the Kruskal–Wallis test between the MWIS-ACO-LS, 1NN, and MWIS on the datasets (classification accuracy).

**Figure 18:**
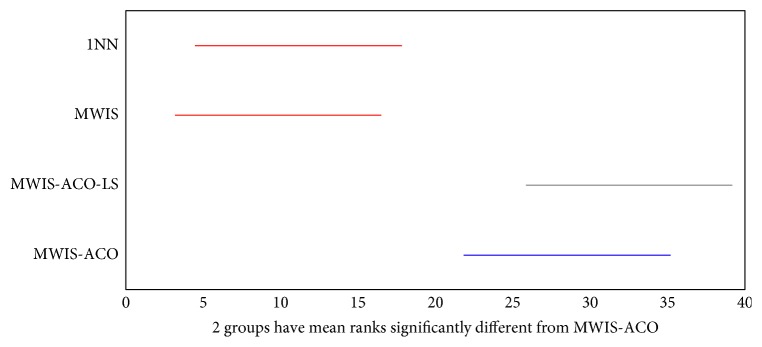
The result of the Kruskal–Wallis test between the MWIS-ACO, 1NN, and MWIS on the datasets (classification accuracy).

The Kruskal–Wallis statistical test presented in Figures 17 and 18 shows a comparison of the results obtained by MWIS, MWIS-ACO, MWIS-ACO-LS, and 1NN classifier. According to these figures, the performance of MWIS-ACO and MWIS-ACO-LS approaches exceeds that of the MWIS method and the 1NN classifier. In terms of the statistical significance of the results (classification accuracy), the said test proves that the difference between the classification accuracy in (“1NN,” “MWIS”) and “MWIS-ACO-LS” is statistically significant (remarkable).

Table 7 lists the best subset of genes selected by our proposed approach (MWIS-ACO-LS) for the datasets in which MWIS-ACO-LS gives the best performances compared to the other works (9_Tumors, Brain_Tumor1, Brain_Tumor2, and Prostate_Tumor). These genes are potential biomarkers in cancer identification.

Based on the experiments we carried out, we can conclude that our approach of gene selection (MWIS-ACO-LS) is well-founded. Indeed, of the ten datasets used, our method has achieved a high classification accuracy. More exactly, the proposed method yielded a classification accuracy equal to or greater than 99.42% for all datasets, with a perfect classification (100%) for 9_Tumors, Brain_Tumor1, Brain_Tumor2, Leukemia1, Leukemia2, SRBCT, Prostate_Tumor, and DLBCL using less than 40 genes. The high classification accuracy found by our proposed methodology returns to two elements: the first is the combination of a method of the graph theory (MWIS) and the ACO metaheuristic, and the second is the use of a modified 1NN classifier where we normalize the training data in order to transform it to a common scale.

In the following, we do a comparison between our proposed method and some recent optimization algorithms using several classification datasets.

### 4.1. Comparison with State-of-the-Art Algorithms

In this section, we compare our method with eight recently referred algorithms in the literature [6, 21–25, 28]. And to make sense of this comparison, the experiments are performed under the same conditions in each algorithm. Specifically, our approach is executed ten times on each dataset, and then we choose the average and the best subset of genes found. We indicate that the papers [22, 23] report just the best results found.

Table 8 summarizes the classification accuracy and the number of selected genes (taken from the original papers) for the different approaches. The (−) symbol indicates that the result is not reported in the related work. We remark that the results obtained by our approach are very encouraging compared to previous work. Indeed, for most of the datasets examined, the classification accuracies obtained by the proposed gene selection method matched or outperformed those obtained using other methods [6, 21–25, 28].

First, for the dataset (9_Tumors) we achieve a perfect accuracy classification with only 40 genes. We find that the best performance for this dataset is attained by our approach (MWIS-ACO-LS), exceeding the best-known result by 5% in the accuracy [6]. We note that the number of genes reported in the FBPSO-SVM [6] is 71 genes to have a good accuracy.

Similarly, for the datasets (11_Tumors, Brain_Tumor1, Brain_Tumor2, and Prostate_Tumor), we get the best performance. In addition, we have a perfect classification (100%) for (Brain_Tumor1, Brain_Tumor2, and Prostate_Tumor) with less than 21 genes.

Table 9 reports the rank of the proposed method compared to other existing methods according to the average accuracy. The results mentioned in the table show that the proposed method has achieved the best average accuracy in most datasets. Indeed, we clearly see that our method is more suitable for gene selection. As shown in Tables 8 and 9, we match or exceed the performance of all comparison methods; except for the Brain_Tumor2 and Prostate_Tumor datasets, in which our approach comes in the second rank after the FBPSO-SVM approach.

The results of this comparative analysis with previous methods for the gene selection in the context of cancer classification have enabled us to conclude that our nature-inspired optimization method is useful in the gene selection problem.

## 5. Conclusion

In this work, we have presented a hybrid approach (MWIS-ACO-LS) for the gene selection in DNA microarray data. The two-stage proposed approach consists of a preselection phase carried out using a new graph-theoretic approach to select first a small subset of genes; in this stage, we model the gene selection problem as an MWIS problem, and we present a greedy algorithm to approximate the MWIS of genes and a search phase that determines a near-optimal subset of genes for the cancer classification. The latter is based on a modified ACO and a LS algorithm.

This approach aims to select a small subset of relevant genes from an original dataset which contains redundant, noisy, or irrelevant data.

The experimental results show that our approach compares very favorably with the reference methods in terms of the classification accuracy and the number of selected genes. Although the results obtained are interesting and encouraging, many points are likely to be studied in future works, such as

- Modifying the MWIS method in order to improve the quality of the first subset of genes
- Combining the MWIS filter with other metaheuristics such as VNS

This field of research will always remain active as long as it is motivated by the advances of data collection and storage systems on one hand, and by the oncology requirements on the other hand. The best approach for judging this selection of genes is to collaborate with experts (biologists) for a good interpretation of the results.

## Figures and Tables

**Algorithm 1:**
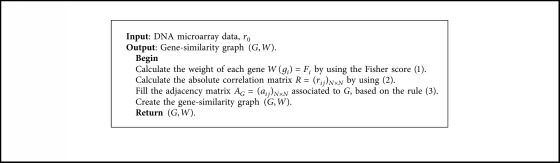
Construction of a gene-similarity graph.

**Algorithm 2:**
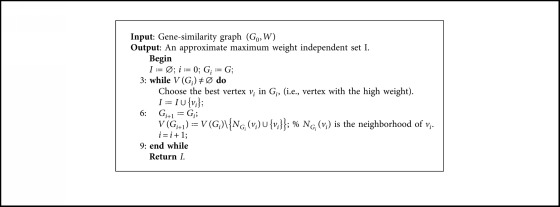
Greedy algorithm to approximate the MWIS.

**Algorithm 3:**
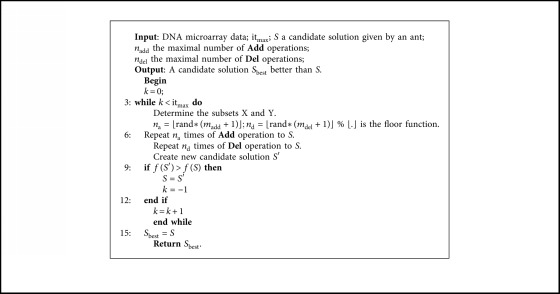
Local search algorithm for gene selection.

**Algorithm 4:**
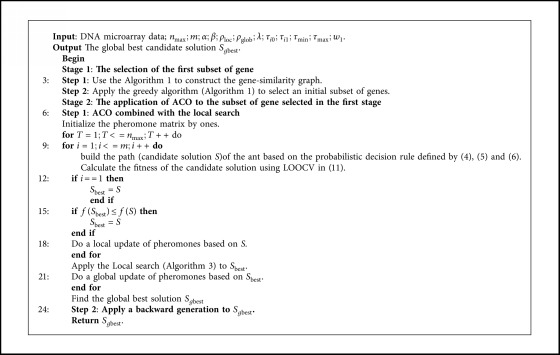
Proposed approach (MWIS-ACO-LS).

**Table 1:** A gene expression matrix composed with *M* samples and *N* genes.

Gene_id_	Gene_1_	Gene_2_	⋯	Gene_*N*_	Class label
Sample_1_	*x*_11_	*x*_12_	⋯	*x*_1*N*_	*y*_1_
Sample_2_	*x*_21_	*x*_22_	⋯	*x*_2*N*_	*y*_2_
Sample_3_	*x*_31_	*x*_32_	⋯	*x*_3*N*_	*y*_3_
⋮	⋮	⋮	⋱	⋮	⋮
Sample_*M*_	*x*_*M*1_	*x*_*M*2_	⋯	*x*_*MN*_	*y*_*M*_

**Table 2:** Correlation (similarity) matrix.

	*g*_1_	*g*_2_	*g*_3_	*g*_4_	*g*_5_	*g*_6_	*g*_7_
*g*_1_	1	0.59	0.19	0.45	0.1	0.24	0.67
*g*_2_	0.59	1	0.36	0.3	0.11	0.07	0,66
*g*_3_	0.19	0.36	1	0.31	0.24	0.06	0.29
*g*_4_	0.45	0.3	0.31	1	0.49	0.81	0.12
*g*_5_	0.1	0.11	0.24	0.49	1	0.72	0.66
*g*_6_	0.24	0.07	0.06	0.81	0.72	1	0.57
*g*_7_	0.67	0.66	0.29	0.12	0.66	0.57	1

**Table 3:** Adjacency matrix.

	*g*_1_	*g*_2_	*g*_3_	*g*_4_	*g*_5_	*g*_6_	*g*_7_
*g*_1_	0	1	0	1	0	0	1
*g*_2_	1	0	1	0	0	0	1
*g*_3_	0	1	0	0	0	0	0
*g*_4_	1	0	0	0	1	1	0
*g*_5_	0	0	0	1	0	1	1
*g*_6_	0	0	0	1	1	0	1
*g*_7_	1	1	0	0	1	1	0

**Table 4:** Parameters used for experiments (common parameters for MWIS-ACO-LS).

Common parameters for MWIS		Value
*r*_0_	The correlation for crating a vertex	0.35

Common parameters for ACO		

m	Population size	30
n_max_	The number of iteration	100
*α*	Influence of the pheromone	1.2
*β*	Heuristic information	0.2
*ρ*_loc_	Local evaporation of pheromone	0.002
*ρ*_glob_	Local evaporation of pheromone	0.06
*λ*	Factor of updating pheromone	1.6
*τ*_*i*0_	The initial pheromone of pathway 0	1.0
*τ*_*i*1_	The initial pheromone of pathway 1	1.0
*τ*_min_	The lower pheromone	0.05
*τ*_max_	The upper pheromone	1.4
*w*_1_	The weight coefficient in the fitness function	0.99

Common parameter for the local search algorithm		

it_max_	The maximal number of iteration without improvement	5
*n*_add_	The maximal number of **Add** operations	3
*n*_del_	The maximal number of **Del** operations	5

Common parameter for the backward generation algorithm		

it_max_	The maximal number of iteration without improvement	20
*n*_add_	The maximal number of **Add** operations	0
*n*_del_	The maximal number of **Del** operations	2

Common parameter for the KNN classifier		

*K*	The number of neighbors	1
The distance used	The Euclidean distance	

Common parameters for the logistic regression model (LR)		

*α*_LR_	The regularization term constant	0.0001
*l*1_ratio_	The weight given to *L*_1_ into the Elasticnet	0.5

**Table 5:** Description of the datasets (DNA microarray) used.

Dataset name	Diagnostic task	Number of samples	Number of genes	Number of classes
11_Tumors	11 various human tumor types	174	12533	11
9_Tumors	9 various human tumor types	60	5726	9
Brain_Tumor1	5 human brain tumor types	90	5920	5
Brain_Tumor2	4 malignant glioma types	50	10367	4
Leukemia1	Acute myelogenous leukemia (AML), acute lympboblastic leukemia (ALL) B-cell, and ALL T-cell	72	5327	3
Leukemia 2	AML, ALL, and mixed-lineage leukemia (MLL)	72	11225	3
Lung_Cancer	4 lung cancer types and normal tissues	203	12600	5
SRBCT	Small, round blue cell tumors (SRBCT) of childhood	83	2308	4
Prostate_Tumor	Prostate tumor and normal tissues	102	10509	2
DLBCL	Diffuse large B-cell lymphomas (DLBCL) and follicular lymphomas	77	5469	2

**Table 6:** Comparison of SVM, 1NN, MWIS-1NN, and MWIS-ACO-LS (LOOCV).

Datasets	Performance	SVM	LR-*L*_1_	Avg	Best	LR-Elasticnet	Avg	1NN	MWIS	MWIS-ACO	MWIS-ACO-LS	Best
11_Tumors	Accuracy (%)	85.63	88.22	93.1	86.38	88,22	74,14	67,24	94,90	96	99,14	**99,42**
	Genes	12533	—	—	—	—	12533	1308	463,00	460	166,9	**101**
	Time (min)	—	—	—	—	—	—	0,82	91,33	—	123,2	—

9_Tumors	Accuracy (%)	38.33	35.00	50.00	29.50	38,33	40,00	60,00	98,83	**100**	**100,00**	**100,00**
	Genes	5726	—	—	—	—	5726	263	90,10	83	51	**40**
	Time (min)	—	—	—	—	—	—	0,34	21,3	—	34,48	—

Brain_Tumor1	Accuracy (%)	88.89	85.67	88.89	85.44	88,89	85,56	80,00	96,56	**100,00**	99,22	**100,00**
	Genes	5920	—	—	—	—	5920	246	55,90	46	22,9	**19**
	Time (min)	—	—	—	—	—	—	0,22	29,14	—	45,81	—

Brain_Tumor2	Accuracy (%)	70.00	27.20	32.00	29.20	36,00	60,00	48,00	95,40	**100,00**	99,40	**100,00**
	Genes	10367	—	—	—	—	10367	110	22,40	18	11,1	**11**
	Time (min)	—	—	—	—	—	—	0,55	17,89	—	27,13	—

Leukemia1	Accuracy (%)	97.22	91.81	94.44	92.64	95,83	83,33	66,67	**100,00**	**100,00**	**100,00**	**100,00**
	Genes	5327	—	—	—	—	5327	297	63,00	56	9,4	**5**
	Time (min)	—	—	—	—	—	—	0,26	25,94	—	43,77	—

Leukemia2	Accuracy (%)	97.22	91.81	95,83	91.67	95.83	86.11	73.61	**100.00**	**100.00**	**100.00**	**100.00**
	Genes	11225	—	—	—	—	11225	203	45.80	42	13.9	**11**
	Time (min)	—	—	—	—	—	—	0,44	23,85	—	37.67	—

Lung_Cancer	Accuracy (%)	95.07	91.67	94.10	91.03	93.60	87.68	90.15	98.42	99.01	98.92	**99.51**
	Genes	12600	—	—	—	—	12600	602	180,00	183	34,8	**36**
	Time (min)	—	—	—	—	—	—	0,82	74,61	—	107,3	—

SRBCT	Accuracy (%)	**100**.**00**	97.59	**100**.**00**	97.10	98.79	85,54	91,57	**100,00**	**100,00**	**100,00**	**100,00**
	Genes	2308	—	—	—	—	2308	109	15,60	15	7,6	**6**
	Time (min)	—	—	—	—	—	—	0,14	23,48	—	38,24	—

Prostate_Tumor	Accuracy (%)	92.16	81.47	88.23	80.69	83.33	82,35	79,41	98,24	99,04	99,12	**100,00**
	Genes	10509	—	—	—	—	10509	193	47,30	43	20,3	**21**
	Time (min)	—	—	—	—	—	—	0,39	29,2	—	46,59	—

DLBCL	Accuracy (%)	97.40	91.95	97.40	91.43	94.80	84,42	87,01	**100,00**	**100,00**	**100,00**	**100,00**
	Genes	5469	—	—	—	—	5469	147	25,7	22	7,2	**6**
	Time (min)	—	—	—	—	—	—	0,17	27,18	—	43,63	—

Note: the best results are shown in bold. Remark: as the SVM, 1NN, and MWIS are of deterministic nature, the classification is calculated just in one run. Accuracy: the classification accuracy using LOOCV (leave-one-out-cross-validation). Genes: the number of genes used in the classification ofthe LR-*L*_1_ and LR-Elasticnet methods. Best: the best result found in all ten runs. Avg: the average of the ten experiments. Time: the execution time in minutes. SVM: the support vector machine classifier using a linear kernel. LR-*L*_1_: the logistic regression classifier with the lasso regularisation. LR-Elasticnet: the logistic regression classifier with the elastic net regularisation. 1NN: the 1-nearest neighbor classifier. MWIS: the maximum weight independent set for gene selection. MWIS-ACO: our method of selection combining MWIS and ACO without using LS. MWIS-ACO-LS: our improved method of selection combining MWIS and ACO and the local search algorithm (LS).

**Table 7:** List of selected genes using MWIS-ACO-LS.

Dataset name	Selected genes' gene no.
9_Tumors	79; 139; 507; 552; 712; 1297; 1364; 1387; 1432; 1444;
	1858; 1952; 1967; 1999; 2022; 2058; 2448; 2498; 2989; 3114;
	3370; 3473; 3662; 3860; 3876; 3922; 3991; 4018; 4050; 4081;
	4525; 4688; 4724; 4783; 4967; 5175; 5265; 5282; 5550; 5698
Brain_Tumor1	26; 318; 868; 895; 925; 1509; 1880; 1950; 2703; 2704;
	3789; 3941; 4269; 4690; 5291; 5342; 5404; 5604; 5746
Brain_Tumor2	1026; 3184; 4049; 4304; 4524; 4639; 4649; 5547; 6892; 8134; 8482
Prostate_Tumor	364; 1478; 1936; 2409; 2445; 2968; 3738; 4032; 4413; 4441;
	4680; 4823; 4960; 5714; 6773; 7437; 8009; 8531; 8574; 9070; 10108

**Table 8:** A comparison between our method (MWIS-ACO-LS) and methods of state-of-the-art.

Datasets	Method	MWIS-ACO-LS	RBPSO-1NN	FBPSO-SVM	FRBPSO	HICATS	EPSO	TS-BPSO	IBPSO	IBPSO
		This work	2018	2018	2017	2016	2013	2009	2008	2011
	Performances		[6]	[6]	[21]	[28]	[25]	[23]	[22]	[24]	
11_Tumors	Best #Acc (%)	**99.42**	—	—	—	97.70	96.55	97.35	93.10	95.4
	Best #Genes	**101**	—	—	—	287	243	3206	2948	228
	Average #Acc (%)	99.14 <1>	—	—	—	95.86	95.40	—	—	95.06
	Average #Genes	166.9	—	—	—	307.5	237.70	—	—	240.9

9_Tumors	Best #Acc (%)	**100.00**	83.33	95.00	—	83.33	76.67	81.63	78.33	78.33
	Best #Genes	40	**20**	71	—	259	251	2941	1280	248
	Average #Acc (%)	**100.00 <1>**	81.83	92.222	—	78.33	75.00	—	—	75.5
	Average #Genes	51	29.1	45	—	248.5	247.10	—	—	240.6

Brain_Tumor1	Best #Acc (%)	**100.00**	94.44	97,78	—	94.44	93.33	95.89	94.44	93.33
	Best #Genes	19	11	21	—	**6**	8	2913	754	5
	Average #Acc (%)	99.22 <1>	94.00	97.22	90.67	93.10	92.11	—	—	92.56
	Average #Genes	22.9	24.7	22.4	803	8.9	7.5	—	—	11.2

Brain_Tumor2	Best #Acc (%)	**100.00**	96.00	**100.00**	—	94.00	94.00	92.65	94.00	94.00
	Best #Genes	11	15	12	—	**3**	4	5086	1197	4
	Average #Acc (%)	99.40 <2>	92.80	**100.00**	87.6	92.60	92.4	—	—	91.00
	Average #Genes	11.1	24.5	14.3	662	5.8	6.0	—	—	6.4

Leukemia1	Best #Acc (%)	**100.00**	**100.00**	**100.00**	—	**100.00**	**100.00**	**100.00**	**100.00**	**100.00**
	Best #Genes	5	8	6	—	3	**2**	2577	1034	**2**
	Average #Acc (%)	**100.00 <1>**	99.72	**100.00**	98.89	**100.00**	**100.00**	—	—	**100.00**
	Average #Genes	9.4	11.7	8.4	825	3	3.2	—	—	3.2

Leukemia2	Best #Acc (%)	**100.00**	**100.00**	**100.00**	—	**100.00**	**100.00**	**100.00**	**100.00**	**100.00**
	Best #Genes	11	5	6	—	5	**4**	5609	1292	**4**
	Average #Acc (%)	**100.00 <1>**	**100.00**	**100.00**	97.50	**100.00**	**100.00**	—	—	**100.00**
	Average #Genes	13.9	13.1	8.6	1028	6.80	6.8	—	—	6.7

Lung_Cancer	Best #Acc (%)	99.51	—	—	—	97.04	96.06	**99.52**	96.55	96.55
	Best #Genes	36	—	—	—	7	7	6958	1897	10
	Average #Acc (%)	98.92 <1>	—	—	—	96.16	95.67	—	—	95.86
	Average #Genes	34.8	—	—	—	7.8	8.3	—	—	14.9

SRBCT	Best #Acc (%)	**100.00**	**100.00**	**100.00**	—	**100.00**	**100.00**	**100.00**	**100.00**	**100.00**
	Best #Genes	**6**	7	10	—	9	7	1084	431	**6**
	Average #Acc (%)	**100.00 <1>**	**100.00**	**100.00**	98.19	**100.00**	99.64	—	—	**100.00**
	Average #Genes	7.6	11.7	12.4	213	11.7	14.90	—	—	17.5

Prostate_Tumor	Best #Acc (%)	**100.00**	99.02	**100.00**	—	98.04	99.02	95.45	92.61	98.04
	Best #Genes	21	9	6	—	**5**	**5**	5320	1294	7
	Average #Acc (%)	99.12 <2>	98.24	**100.00**	92.43	97.75	97.84	—	—	97.94
	Average #Genes	20.3	11.2	8.3	418	7.2	6.6	—	—	13.6

DLBCL	Best #Acc (%)	**100.00**	**100.00**	**100.00**	—	**100.00**	**100.00**	**100.00**	**100.00**	**100.00**
	Best #Genes	6	6	4	—	**3**	**3**	2671	1042	4
	Average #Acc (%)	**100.00 <1>**	**100.00**	**100.00**	96.49	100.00	100.00	—	—	**100.00**
	Average #Genes	7.2	12.5	6.7	105	4.10	4.70	—	—	6

< >: the rank of our method in a specific average accuracy. RBPSO-1NN = a gene selection method based on the combination of ReliefF and BPSO and 1NN as a classifier. FBPSO-SVM = a gene selection method based on the combination of Fisher score and BPSO and the SVM as a classifier; FRBPSO = a fuzzy rule based binary PSO; HICATS=Hybrid Binary Imperialist Competition Algorithm and Tabu Search; EPSO = an enhancement of binary particle swarm optimization; TS-BPSO = A combination of tabu search and BPSO; IBPSO = an improved binary PSO.

**Table 9:** Rank of our method comparatively to the existing methods according to the average accuracy.

Dataset	Method						
	MWIS-ACO-LS	RBPSO-1NN	FBPSO-SVM	FRBPSO	HICATS	EPSO	IBPSO
	Our proposed method						
11_Tumors	**1**	—	—	—	2	3	4
9_Tumors	**1**	3	2	—	4	6	5
Brain_Tumor1	**1**	3	2	7	4	6	5
Brain_Tumor2	2	3	**1**	7	4	5	6
Leukemia1	**1**	3	**1**	2	**1**	**1**	**1**
Leukemia2	**1**	**1**	**1**	2	**1**	**1**	**1**
Lung_Cancer	**1**	—	—	—	2	4	3
SRBCT	**1**	**1**	**1**	3	**1**	2	**1**
Prostate_Tumor	2	3	**1**	7	6	5	4
DLBCL	**1**	**1**	**1**	2	**1**	**1**	**1**

## Data Availability

The datasets (DNA microarray) used in our paper are publicly available and easily accessible [62] (http://web.archive.org/web/20180625075744/http://www.gems-system.org/) (visited on 2018).
